# Web-Delivered Cognitive Behavioral Therapy for Distressed Cancer Patients: Randomized Controlled Trial

**DOI:** 10.2196/jmir.8850

**Published:** 2018-01-31

**Authors:** Suzanne K Chambers, Lee M Ritterband, Frances Thorndike, Lisa Nielsen, Joanne F Aitken, Samantha Clutton, Paul A Scuffham, Philippa Youl, Bronwyn Morris, Peter D Baade, Jeff Dunn

**Affiliations:** ^1^ Menzies Health Institute Queensland Griffith University Gold Coast Australia; ^2^ Cancer Council Queensland Brisbane Australia; ^3^ Prostate Cancer Foundation of Australia Sydney Australia; ^4^ Health and Wellness Institute Edith Cowan University Perth Australia; ^5^ Centre for Clinical Research The University of Queensland Brisbane Australia; ^6^ University of Virginia Charlottesville, VA United States; ^7^ BeHealth Solutions Charlottesville, VA United States; ^8^ Institute for Resilient Regions University of Southern Queensland Springfield Australia; ^9^ University of Sunshine Coast Sippy Downs Australia; ^10^ School of Public Health and Social Work Queensland University of Technology Brisbane Australia; ^11^ Menzies School of Health Research Darwin Australia; ^12^ School of Mathematical Sciences Queensland University of Technology Brisbane Australia; ^13^ School of Social Science The University of Queensland Brisbane Australia

**Keywords:** cancer, mental health, psychological distress, randomized controlled trial (RCT), health services delivery

## Abstract

**Background:**

Web-based interventions present a potentially cost-effective approach to supporting self-management for cancer patients; however, further evidence for acceptability and effectiveness is needed.

**Objective:**

The goal of our research was to assess the effectiveness of an individualized Web-based cognitive behavioral therapy (CBT) intervention on improving psychological and quality of life outcomes in cancer patients with elevated psychological distress.

**Methods:**

A total of 163 distressed cancer patients (111 female, 68.1%) were recruited through the Queensland Cancer Registry and the Cancer Council Queensland Cancer Helpline and randomly assigned to either a Web-based tailored CBT intervention (CancerCope) (79/163) or a static patient education website (84/163). At baseline and 8-week follow-up we assessed primary outcomes of psychological and cancer-specific distress and unmet psychological supportive care needs and secondary outcomes of positive adjustment and quality of life.

**Results:**

Intention-to-treat analyses showed no evidence of a statistically significant intervention effect on primary or secondary outcomes. However, per-protocol analyses found a greater decrease for the CancerCope group in psychological distress (*P*=.04), cancer-specific distress (*P*=.02), and unmet psychological care needs (*P*=.03) from baseline to 8 weeks compared with the patient education group. Younger patients were more likely to complete the CancerCope intervention.

**Conclusions:**

This online CBT intervention was associated with greater decreases in distress for those patients who more closely adhered to the program. Given the low costs and high accessibility of this intervention approach, even if only effective for subgroups of patients, the potential impact may be substantial.

**Trial Registration:**

Australian New Zealand Clinical Trials Registry ACTRN12613001026718; https://www.anzctr.org.au/Trial/Registration/TrialReview.aspx?id=364768&isReview=true (Archived by WebCite at http://www.webcitation.org/6uPvpcovl)

## Introduction

In 2012 it was estimated that there were 14.1 million new cases of cancer diagnosed globally [1]. Estimates suggest that in 2030 this number will reach 21.6 million [1], a substantial increase in the cancer burden that will in turn increase demands on the health care system. In this regard, people affected by cancer frequently report heightened psychological distress [2] that for some persists for many years [3-5]. It is now well acknowledged that psychosocial care is an essential component of quality cancer care [6]. However, how to deliver evidence-based psychosocial care on a population basis, given the current and future predicted prevalence of cancer and increasingly limited health care resources, remains a challenge.

Approaches to more effectively deliver evidence-based psychosocial care include a low-intensity framework through which cost-effective services can be delivered. Low-intensity care models have as their guiding values the principles of equity and access, with tailoring to the extent and depth of need and use of flexible delivery methods [7]. Within this framework, self-management has been proposed as an effective method by which patient needs can be met [8,9]. Web-based interventions present a specific appeal here as a remotely delivered low-cost approach to supporting self-management with potential for widespread dissemination [10]. Indeed, Web-based programs have been found to be effective in promoting behavior change with regard to stress management, exercise, nutrition, and participation in health care [11,12]. There are, however, questions still to be answered about the acceptability and effectiveness of Web-based interventions to improve psychological outcomes for cancer patients.

Accordingly, we undertook a randomized controlled trial to assess the effectiveness of an individualized Web-based cognitive behavioral intervention (CancerCope) in cancer patients who have or are at risk of having elevated psychological distress. CancerCope was compared with a static patient education website with participants assessed over a 2-month period. We hypothesized that, relative to participants receiving patient education, participants receiving CancerCope would have lower psychological and cancer-specific distress, lower unmet psychological supportive care needs, higher positive adjustment, and improved quality of life.

## Methods

### Participants

Participants were recruited through the Queensland Cancer Registry (QCR), a population-based register of cancer diagnoses in Queensland, and the Cancer Council Queensland Cancer Helpline, a telephone information and support service. Eligible participants were adults who had been diagnosed with cancer who scored ≥4 on the Distress Thermometer [13] (indicating high distress or risk of high distress); were able to read and speak English; had no history of head injury, dementia or psychiatric illness; had no concurrent cancer; and had phone and Internet access. Participants recruited through the QCR had 2 additional eligibility criteria: consent from their diagnosing clinician to participate and having been diagnosed with melanoma or colorectal cancer within the last 6 months.

### Intervention

Participants in the intervention arm were provided access to the CancerCope program, an online support program based on a 5-session telephone-based cognitive behavioral therapy intervention [8,14] and modified to include 6 cores covering: the cancer journey, understanding stress, managing worry, tackling problems, taking care (improving well-being), and moving forward. The cores consisted of educational information and expert videos from psychologists as well as stories and videos of 4 fictional characters on their cancer journey as a way to illustrate the different experiences of others. The program had high levels of interactivity to increase user engagement and systems to encourage use and self-management including personalized email reminders and feedback. Content was tailored in response to the participant’s needs as determined by their input, including assigned behavioral homework supported by the interactive components of the website. For example, users received tailored feedback based on distress scores and concerns. Users were also able to set personal goals and receive recommended goals. These were then tracked throughout the use of the program and could be modified by the user as needed.

Components that targeted challenges associated with cancer treatments (eg, pain, sleep disturbance, fatigue) were additionally selected if relevant. Cores were completed weekly over a 6-week period rather than available all at once, with ongoing access to the program provided for 12 months. Cores were marked as completed if the participant manually submitted them as complete. Screenshots of the CancerCope program can be found in Multimedia Appendix 1. A more detailed description of the program has been published elsewhere [15].

The control condition was a static patient education website containing information covering stress management skills, problem-solving approaches to cancer-related concerns, and patient education about a healthy lifestyle to promote wellness and optimize quality of life.

Participants were provided with the URL for the study website and a unique username and password that gave them individualized access to the program. Only the research team (project manager and staff involved with recruitment and follow-up) had access to participant information (including name and contact details) through a secure password-protected database. Data collected through online questionnaires were downloaded and saved on a secure password-protected server.

### Study Integrity

Ethical approval was obtained from the Griffith University Human Research Ethics Committee (PSY/70/13/HREC) and Metro South Human Research Ethics Committee (HREC/13/QPAH/601). The study was guided by the Consolidated Standards of Reporting Trials (CONSORT) statement [16]. Randomization followed baseline assessment and occurred in blocks of 10, with each condition randomly generated 5 times within each block to ensure an unpredictable allocation sequence with equal numbers of participants in each group at the completion of each block. This sequence was undertaken by the project manager and concealed from investigators. Assessments were through self-report questionnaires. Primary analyses were intention to treat.

### Materials

Baseline assessment was conducted by telephone. Follow-up assessment occurred after the intervention period (8 weeks) via online questionnaires accessed through the Web program.

#### Outcome Measures

Primary outcome measures included the Brief Symptom Inventory 18 [17], the Impact of Event Scale [18,19], and the Supportive Care Needs Survey Short Form 34 [20]. Higher scores on the first 2 measures indicated greater psychological or cancer-specific distress, respectively. Secondary outcome measures were the Posttraumatic Growth Inventory [21] and the Assessment of Quality of Life 8D [22]. Higher scores indicated greater benefit finding or quality of life, respectively. Process measures, as detailed next, were also included for the intervention arm.

#### Process Measures

Participants in the CancerCope condition completed 3 process measures following the 8-week intervention period. The Internet Evaluation and Utility Questionnaire assesses patients’ experiences and perceptions of an Internet intervention [23,24]. The constructs measure ease of use, convenience, engagement, enjoyment, layout, privacy, satisfaction, acceptability, and perceptions of the Web program material in terms of usefulness, comprehension, credibility, likelihood of returning, mode of delivery, and helpfulness. Higher scores indicate more positive experiences and perceptions of the Web program. The Internet Intervention Adherence Questionnaire identifies obstacles and barriers that interfere with using Internet intervention programs [23,25]. Higher scores indicate the participant experienced more problems with the Web program. The Internet Impact and Effectiveness Questionnaire assesses patients’ perceptions of the Internet intervention in terms of the program’s effectiveness in resolving their targeted health condition. Perceived impact is measured in terms of helpfulness, knowledge gains, treatment effectiveness for self, treatment effectiveness for others, long-term effectiveness, quality of life, mood, physical activity, family relationships, peer relationships, social activity, school/work attendance, school/work performance, treatment implementation, goal orientation, confidence in ability to manage the health condition, relapse prevention, and service reduction [23,24]. Higher scores indicate greater impact and effectiveness.

### Statistical Analyses

The study design involved a multivariate, 2-condition randomized controlled trial with repeated measures across time. A hierarchical linear model analysis was used to reflect this design in which measurement occasions (level 1) were nested within persons (level 2) and program differences were represented as a fixed effect at level 2 and the interaction with time suggested differential adjustment and distress trajectories for the 2 groups. The analysis examined the effect of study group (CancerCope and patient education) and time point (baseline and 2 months) on the specific primary and secondary outcome scores, including an interaction term between the 2 variables (study group and time point). We assessed differences in baseline demographic characteristics and baseline measures between respondents who did and did not complete the second questionnaire by performing multivariate backwards stepwise logistic regression analysis.

To facilitate an intention-to-treat analysis, multiple imputation (using 50 imputations) was used to impute missing data for those respondents who completed the baseline but not the follow-up assessment. The multiple imputation process involved regression of the relevant outcome variable with all the nonmissing values of the baseline outcome measures, with the addition of age group and sex. Subsequent statistical commands were run on the imputed data, with the coefficients and standard errors adjusted for the variability between imputations using Rubin’s combination rules [26]. Multiple imputation assumes that the missing data is missing-at-random. However, since poor health may be a contributing factor for noncompletion and withdrawal, we included a sensitivity analysis similar to that suggested by Biering and colleagues [27] to see what impact reducing imputed values by 25% had on the model results.

Per-protocol analyses were conducted by repeating these analyses for those respondents who accessed at least 3 cores of the CancerCope intervention and comparing these respondents to the control respondents. Differences in baseline demographic characteristics and baseline measures between respondents who accessed at least 3 cores and those who accessed fewer than 3 cores were analyzed by performing and reporting the results of multivariate backwards stepwise logistic regression analysis.

Effect sizes for the per-protocol analysis were estimated for each continuous outcome variable based on Cohen *d* [28], with the mean difference scores (baseline to 2-month) being compared between the intervention (at least 3 cores accessed) and the patient education group. Test statistics of Cohen *d* and 95% confidence intervals were run for each imputation separately and then combined across the multiple imputations using Rubin’s rule [29].

A post-hoc power calculation based on 79 people in the CancerCope intervention and 84 in the patient education arm (163 in total) showed our study cohort provided 89% power to detect a medium effect size (0.5) with a significance level (alpha) of .05 using a 2-sided 2-sample *t* test. All analysis was conducted in Stata 15 (StataCorp LLC).

## Results

### Participants

**Figure 1:**
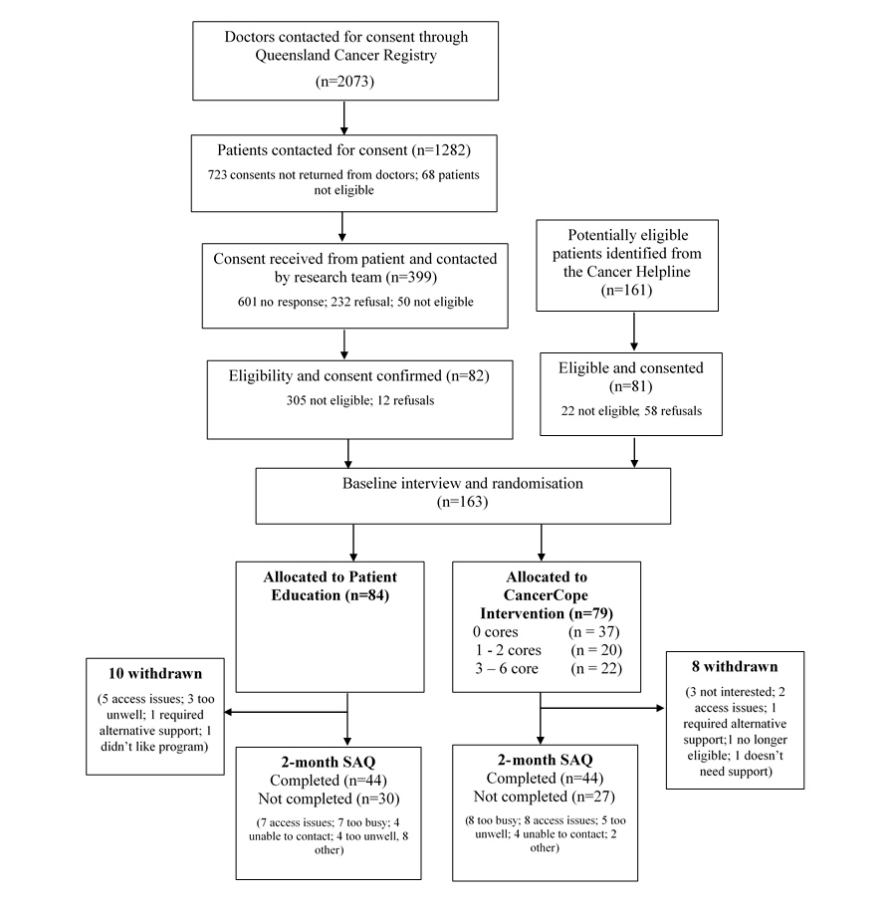
CONSORT flowchart from baseline to 2 months.

Between April 2015 and May 2016, a total of 163 participants were recruited through the QCR and the Cancer Helpline (Figure 1) and randomly assigned to patient education (n=84) or CancerCope intervention (n=79). A detailed description of the sample of this trial has been published elsewhere [15]. In the sample, 68.1% (111/163) were female, the mean age of participants was 57 years, over 60% of the sample (100/163) had completed further education after high school, the most common cancer type was colorectal (60/163, 36.8%) followed by breast (42/163, 25.8%) and melanoma (29/163, 17.8%), and median days since diagnosis was 139. Respondents were more likely to complete the second questionnaire if they were retired rather than employed or other work status (χ^2^_2_=6.8, *P*=.03) or had higher unmet sexuality needs (χ^2^_1_=5.6, *P*=.02) or lower unmet physical needs (χ^2^_1_=4.3, *P*=.04).

Of those in the CancerCope intervention group, 10% (8/79) accessed all 6 cores, with 47% (37/79) not accessing any cores; 28% (22/79) accessed 3 or more cores and were classified as completers. For participants in the patient education group, 55% (46/84) accessed the patient education website content. Of those, 61% (28/46) accessed the site once, 33% (15/46) accessed the site 2 to 4 times, and 6% (3/46) accessed the site 5 or more times.

### Effectiveness

The intention-to-treat analysis (Multimedia Appendix 2) showed no evidence of a statistically significant intervention effect on any of the primary or secondary outcome variables, with these results robust to the missing-at-random assumption (Multimedia Appendix 3). A secondary per-protocol analysis restricted within the CancerCope group to those who accessed at least 3 cores during the study period (n=22) found evidence of a greater decrease in psychological distress (*P*=.03) and cancer-specific distress (*P*=.02) along with unmet psychological needs (*P*=.03) from baseline to 8 weeks compared with the patient education group (Multimedia Appendix 4). Again, these per-protocol results were robust to the missing-at-random assumption (Multimedia Appendix 5).

When comparing the characteristics of patients in the intervention who accessed ≥3 cores against those who accessed fewer than 3 cores, the demographic variables that were retained in the logistic model through the backward selection process were age group (χ^2^_2_=5.4, *P*=.07), sex (χ^2^_1_=2.8, *P*=.10), and work status (χ^2^_2_=9.9, *P*=.01), suggesting that females and younger patients, including younger patients among those who were retired, were slightly more likely to be in the per-protocol group. In addition, there was also some evidence that respondents who had higher unmet information (χ^2^_1_=2.2, *P*=.14) and patient care (χ^2^_1_=3.2, *P*=.08) needs, higher cancer-specific distress (χ^2^_1_=4.7, *P*=.03), and lower posttraumatic growth (χ^2^_1_=4.0, *P*=.05) were more likely to be in the per-protocol group.

**Figure 2:**
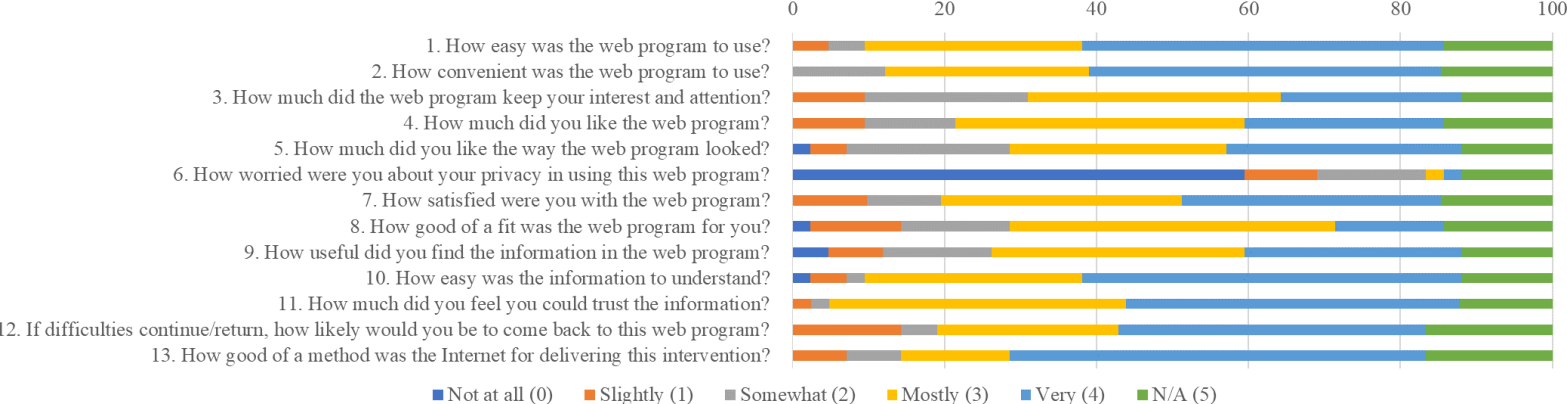
Findings from the Internet Evaluation and Utility Questionnaire (from a response of n=41-42).

**Figure 3:**
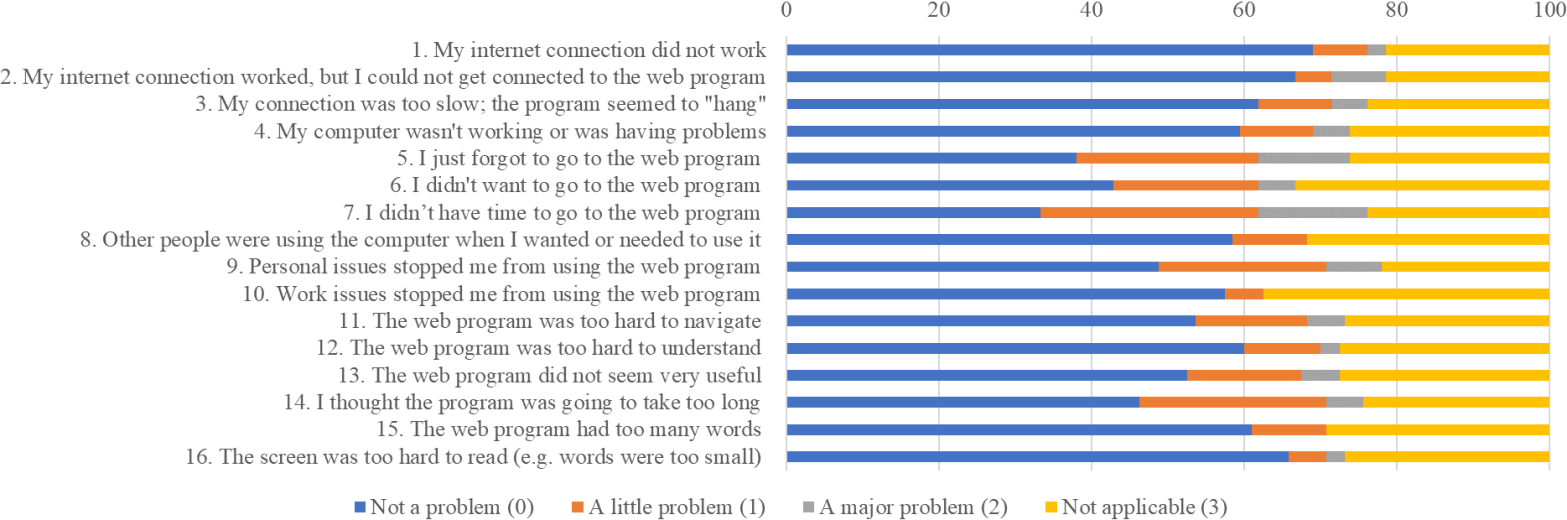
Findings from the Internet Intervention Adherence Questionnaire (from a response of n=40-42).

**Figure 4:**
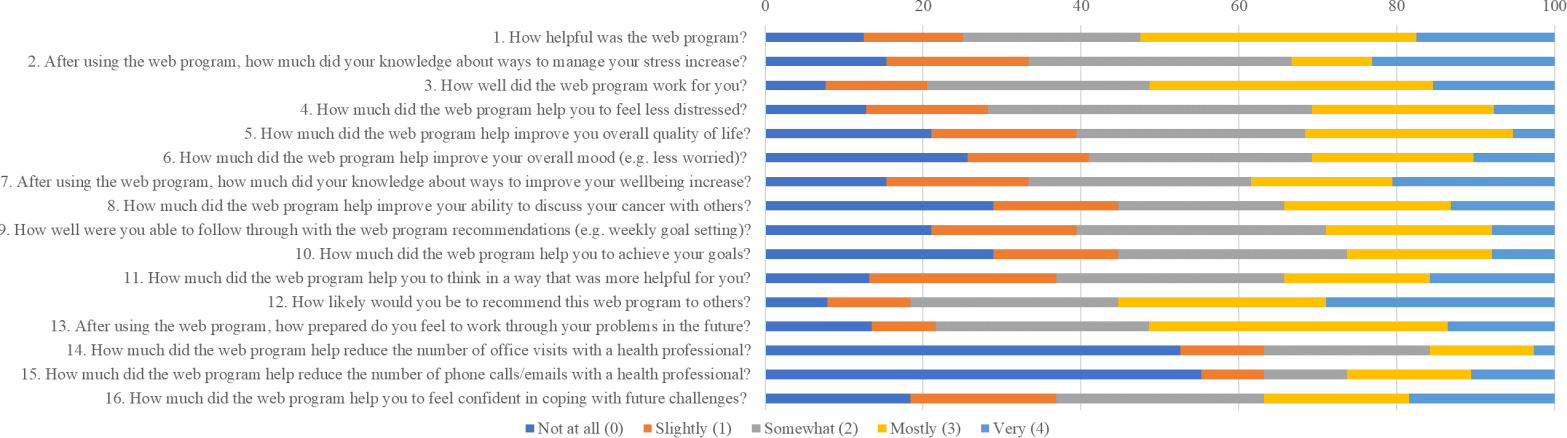
Findings from the Internet Impact and Effectiveness Questionnaire (from a response of n=37-40).

On average, patients in the intervention arm found the CancerCope Web program easy to use, helpful, and a good fit for their needs (Figure 2). The relaxation, meditation, and self-help components were reported as most helpful. Technical problems were infrequent (Figure 3). Patients reported the program as more helpful for improving knowledge, problem solving, and future coping than for mood and would recommend it to others (Figure 4).

## Discussion

Although an intervention effect was not found in the primary analyses, a secondary per-protocol analysis found psychological benefits with medium effects for the subgroup of patients who more closely adhered to the CancerCope program. Hence, while the program overall was positively received by patients, we are not able to conclude it was effective as a standalone psychosocial care intervention. We do, however, have evidence to suggest that if the intervention is used, positive effects can be expected.

The delivery of psychosocial care to cancer patients through a scalable, population-based approach remains an important goal as cancer prevalence increases. The CancerCope program reported in this trial differs from much of the previously reported Web-based psycho-oncology intervention research in that it was a fully automated and tailored intervention and did not include therapist or nurse support or guidance [30], support group forums [31], discussion boards [32,33], or messaging services [34]. Rather, our approach was designed to be completely self-managed by the patient and therefore suitable for widespread dissemination at minimal cost.

One possible way forward may be to view Web-based interventions of this type as an important step in universal psychosocial care within a stepped or tiered model of care [9]. For example, distressed patients or those with unmet psychological care needs could be offered a low-cost self-managed online program such as CancerCope and then stepped or triaged to other more in-depth care models (such as nurse counseling or psychology services) if their distress remains unresolved. Relatedly, and perhaps more efficiently, if we could better identify who might be best served by a Web-based approach as well as who might use the intervention, we could make this type of intervention available to these individuals first. We note that we were not able to recruit our original target sample size and this precluded us from being able to more deeply elucidate the patient subgroups for whom CancerCope was helpful. This is a study limitation. We have previously shown that background variables such as educational level and age moderate the effectiveness of tele-based psychological intervention [35]. Sociodemographic variables such as these may well have influenced participants’ responses to this Web-based intervention; however, our study was not able to clearly examine this possibility. Moving forward, we suggest psychosocial researchers and practitioners in cancer care might consider Web-based programs as a component of stepped care and focus further on what works best and for whom.

## Appendix

Multimedia Appendix 1 Screenshots of the intervention.

Multimedia Appendix 2 Intention-to-treat analysis (baseline vs 2-month) for primary and secondary outcome scores using multiple imputation analysis (50 imputations).

Multimedia Appendix 3 Sensitivity analysis (25% reduction in imputed values): intention-to-treat analysis (baseline vs 2-month) for primary and secondary outcome scores using multiple imputation analysis (50 imputations).

Multimedia Appendix 4 Per-protocol analysis: intention-to-treat analysis (baseline vs 2-month) for primary and secondary outcome scores using multiple imputation analysis (50 imputations).

Multimedia Appendix 5 Sensitivity analysis (25% reduction in imputed values): per-protocol analysis (baseline vs 2-month) for primary and secondary outcome scores using multiple imputation analysis (50 imputations).

Multimedia Appendix 6 CONSORT‐EHEALTH checklist (V 1.6.1).

## Abbreviations

CBT: cognitive behavioral therapy
QCR: Queensland Cancer Registry

## References

[1] Ferlay J, Soerjomataram I, Ervik M, Dikshit R, Esser S, Mathers C, Rebelo M, Parkin DM, Forman D, Bray F Cancer incidence and mortality worldwide: IARC CancerBase—GLOBOCAN 2012 v1.

[2] Zabora J, BrintzenhofeSzoc K, Curbow B, Hooker C, Piantadosi S (2001). The prevalence of psychological distress by cancer site. Psychooncology.

[3] Chambers SK, Ng SK, Baade P, Aitken JF, Hyde MK, Wittert G, Frydenberg M, Dunn J (2017). Trajectories of quality of life, life satisfaction, and psychological adjustment after prostate cancer. Psychooncology.

[4] Dunn J, Ng SK, Breitbart W, Aitken J, Youl P, Baade PD, Chambers SK (2013). Health-related quality of life and life satisfaction in colorectal cancer survivors: trajectories of adjustment. Health Qual Life Outcomes.

[5] Dunn J, Ng SK, Holland J, Aitken J, Youl P, Baade PD, Chambers SK (2013). Trajectories of psychological distress after colorectal cancer. Psychooncology.

[6] Holland J, Watson M, Dunn J (2011). The IPOS new International Standard of Quality Cancer Care: integrating the psychosocial domain into routine care. Psychooncology.

[7] Schofield P, Chambers S (2015). Effective, clinically feasible and sustainable: key design features of psycho-educational and supportive care interventions to promote individualised self-management in cancer care. Acta Oncol.

[8] Chambers SK, Girgis A, Occhipinti S, Hutchison S, Turner J, McDowell M, Mihalopoulos C, Carter R, Dunn JC (2014). A randomized trial comparing two low-intensity psychological interventions for distressed patients with cancer and their caregivers. Oncol Nurs Forum.

[9] Hutchison SD, Steginga SK, Dunn J (2006). The tiered model of psychosocial intervention in cancer: a community based approach. Psychooncology.

[10] Leykin Y, Thekdi SM, Shumay DM, Muñoz RF, Riba M, Dunn LB (2012). Internet interventions for improving psychological well-being in psycho-oncology: review and recommendations. Psychooncology.

[11] Webb T, Joseph J, Yardley L, Michie S (2010). Using the Internet to promote health behavior change: a systematic review and meta-analysis of the impact of theoretical basis, use of behavior change techniques, and mode of delivery on efficacy. J Med Internet Res.

[12] Wantland DJ, Portillo CJ, Holzemer WL, Slaughter R, McGhee EM (2004). The effectiveness of Web-based vs. non-Web-based interventions: a meta-analysis of behavioral change outcomes. J Med Internet Res.

[13] Holland JC, Andersen B, Breitbart WS, Buchmann LO, Compas B, Deshields TL, Dudley MM, Fleishman S, Fulcher CD, Greenberg DB, Greiner CB, Handzo GF, Hoofring L, Hoover C, Jacobsen PB, Kvale E, Levy MH, Loscalzo MJ, McAllister-Black R, Mechanic KY, Palesh O, Pazar JP, Riba MB, Roper K, Valentine AD, Wagner LI, Zevon MA, McMillian NR, Freedman-Cass DA (2013). NCCN clinical practice guidelines in oncology: distress management, version 2.2013. J Natl Compr Canc Netw.

[14] Hutchison SD, Sargeant H, Morris BA, Hawkes AL, Clutton S, Chambers SK (2011). A community-based approach to cancer counselling for patients and carers: a preliminary study. Psychooncology.

[15] Chambers SK, Ritterband L, Thorndike F, Nielsen L, Aitken JF, Clutton S, Scuffham P, Youl P, Morris B, Baade P, Dunn J (2017). A study protocol for a randomised controlled trial of an interactive web-based intervention: CancerCope. BMJ Open.

[16] Altman DG, Schulz KF, Moher D, Egger M, Davidoff F, Elbourne D, Gøtzsche PC, Lang T (2001). The revised CONSORT statement for reporting randomized trials: explanation and elaboration. Ann Intern Med.

[17] Derogatis L, Lopez M (2000). Brief Symptom Inventory 18: Administration, Scoring and Procedures Manual.

[18] Horowitz M, Wilner N, Alvarez W (1979). Impact of Event Scale: a measure of subjective stress. Psychosom Med.

[19] Weiss D, Marmar C, Wilson JP, Keane TM (1997). The Impact of Event Scale–Revised. Assessing Psychological Trauma and PTSD.

[20] Boyes A, Girgis A, Lecathelinais C (2009). Brief assessment of adult cancer patients' perceived needs: development and validation of the 34-item Supportive Care Needs Survey (SCNS-SF34). J Eval Clin Pract.

[21] Tedeschi RG, Calhoun LG (1996). The Posttraumatic Growth Inventory: measuring the positive legacy of trauma. J Trauma Stress.

[22] Richardson J, Iezzi A, Khan MA, Maxwell A (2014). Validity and reliability of the Assessment of Quality of Life (AQoL)-8D multi-attribute utility instrument. Patient.

[23] Ritterband LM, Ardalan K, Thorndike FP, Magee JC, Saylor DK, Cox DJ, Sutphen JL, Borowitz SM (2008). Real world use of an Internet intervention for pediatric encopresis. J Med Internet Res.

[24] Thorndike FP, Saylor DK, Bailey ET, Gonder-Frederick L, Morin CM, Ritterband LM (2008). Development and perceived utility and impact of an Internet intervention for insomnia. E J Appl Psychol.

[25] Ritterband LM, Borowitz S, Cox DJ, Kovatchev B, Walker LS, Lucas V, Sutphen J (2005). Using the internet to provide information prescriptions. Pediatrics.

[26] Rubin D (1987). Multiple Imputation for Nonresponse in Surveys.

[27] Biering K, Hjollund NH, Frydenberg M (2015). Using multiple imputation to deal with missing data and attrition in longitudinal studies with repeated measures of patient-reported outcomes. Clin Epidemiol.

[28] Cohen J (1988). Statistical Power Analysis for the Behavioral Sciences. 2nd Edition.

[29] Rubin DB (1976). Inference and missing data. Biometrika.

[30] Ruland CM, Andersen T, Jeneson A, Moore S, Grimsbø GH, Børøsund E, Ellison MC (2013). Effects of an internet support system to assist cancer patients in reducing symptom distress: a randomized controlled trial. Cancer Nurs.

[31] Wootten AC, Abbott JM, Meyer D, Chisholm K, Austin DW, Klein B, McCabe M, Murphy DG, Costello AJ (2015). Preliminary results of a randomised controlled trial of an online psychological intervention to reduce distress in men treated for localised prostate cancer. Eur Urol.

[32] Owen JE, Klapow JC, Roth DL, Shuster JL, Bellis J, Meredith R, Tucker DC (2005). Randomized pilot of a self-guided internet coping group for women with early-stage breast cancer. Ann Behav Med.

[33] Duffecy J, Sanford S, Wagner L, Begale M, Nawacki E, Mohr DC (2013). Project onward: an innovative e-health intervention for cancer survivors. Psychooncology.

[34] Yun YH, Lee KS, Kim YW, Park SY, Lee ES, Noh DY, Kim S, Oh JH, Jung SY, Chung KW, Lee YJ, Jeong SY, Park KJ, Shim YM, Zo JI, Park JW, Kim YA, Shon EJ, Park S (2012). Web-based tailored education program for disease-free cancer survivors with cancer-related fatigue: a randomized controlled trial. J Clin Oncol.

[35] Chambers SK, Ferguson M, Gardiner RA, Aitken J, Occhipinti S (2013). Intervening to improve psychological outcomes for men with prostate cancer. Psychooncology.

