# Supplementary material for: Web-Delivered Cognitive Behavioral Therapy for Distressed Cancer Patients: Randomized Controlled Trial
Source: J Med Internet Res. 2018 Jan 31;20(1):e42. doi: 10.2196/jmir.8850 (PMC5812983; doi:10.2196/jmir.8850)

Appendix 1. Screenshots of the intervention

Additional screenshots of the CancerCope web program.

Home page

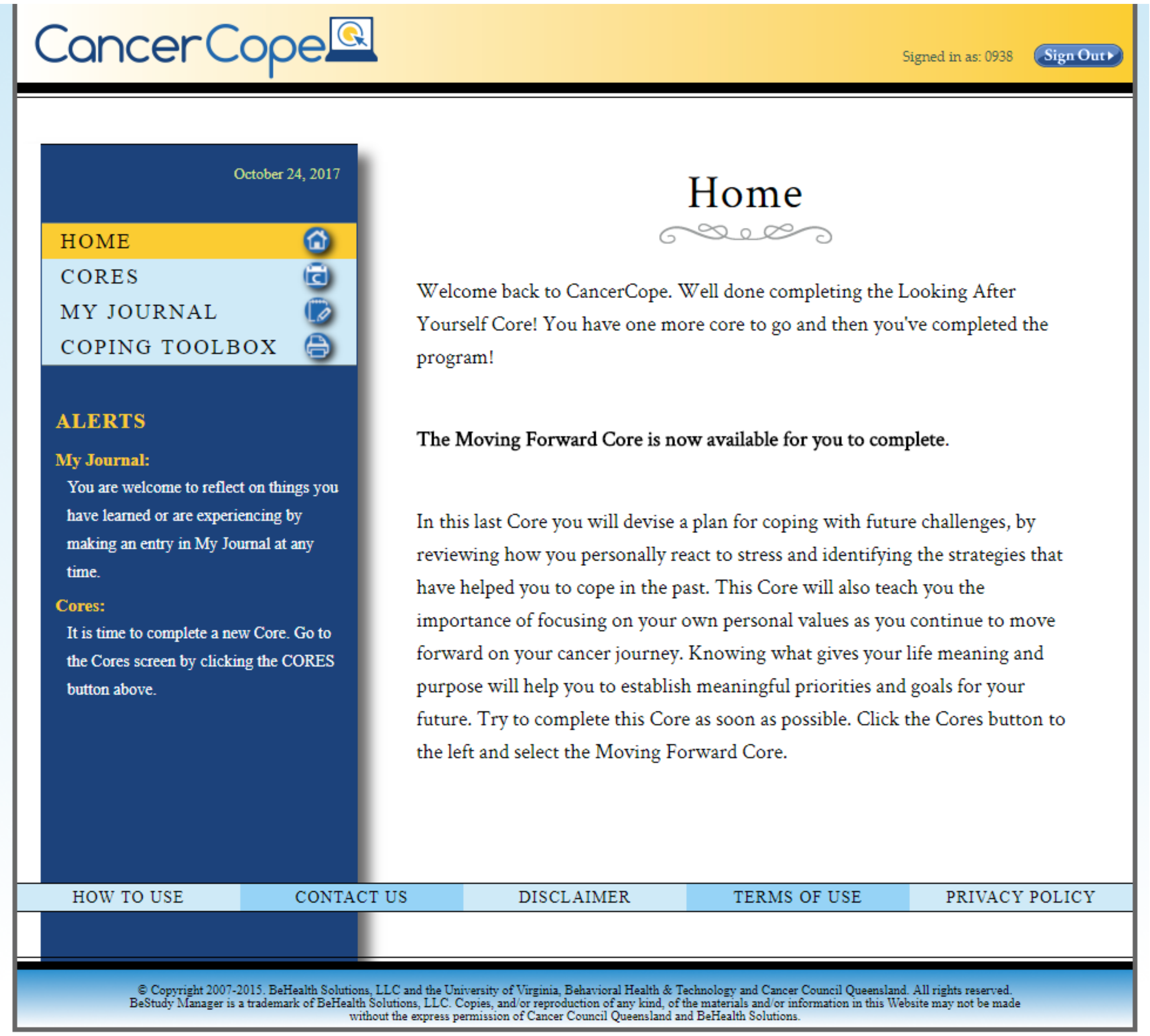

The CancerCope program has six cores for participants to complete

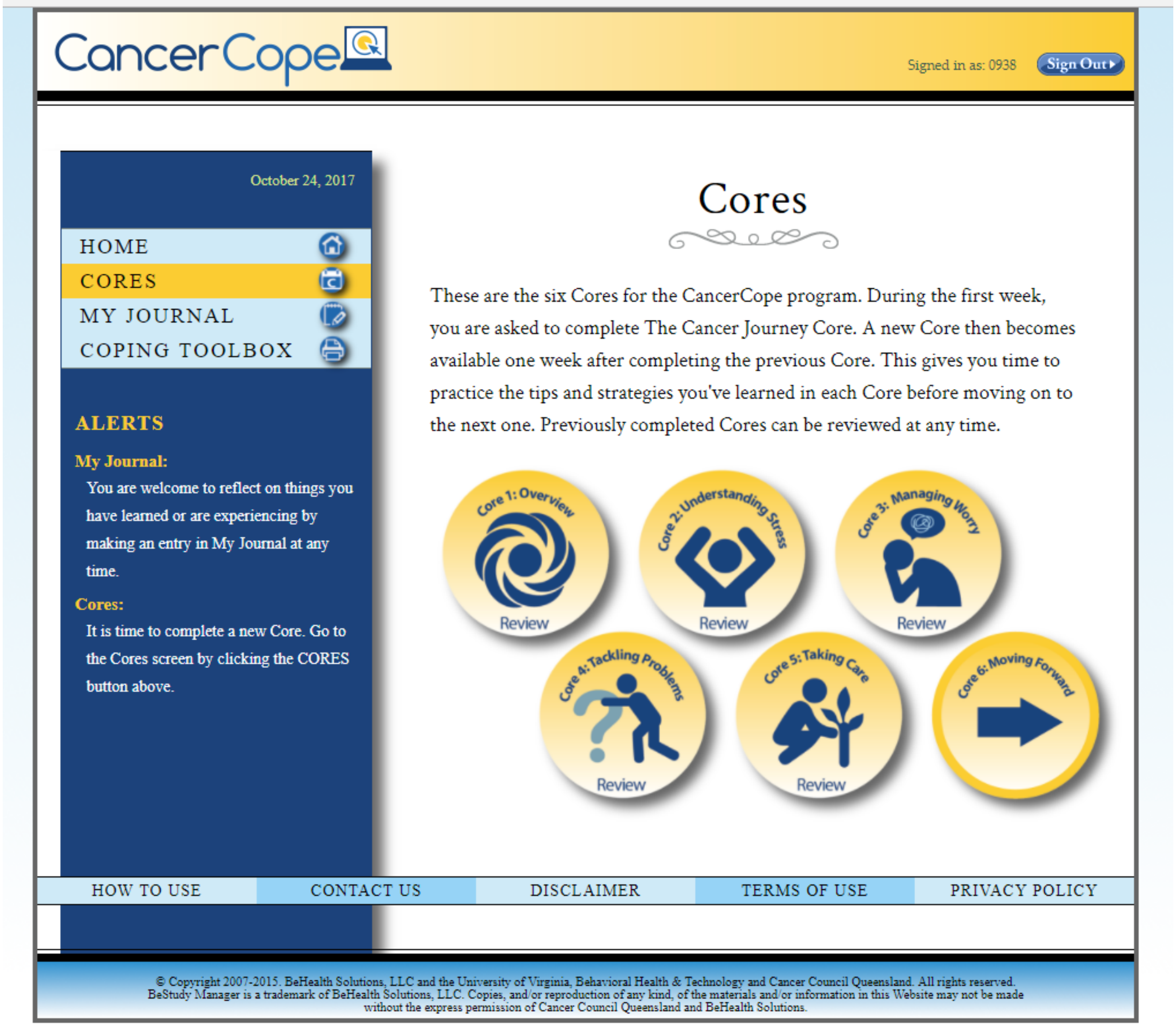

The CancerCope program has high levels of interactivity to encourage use and self-management

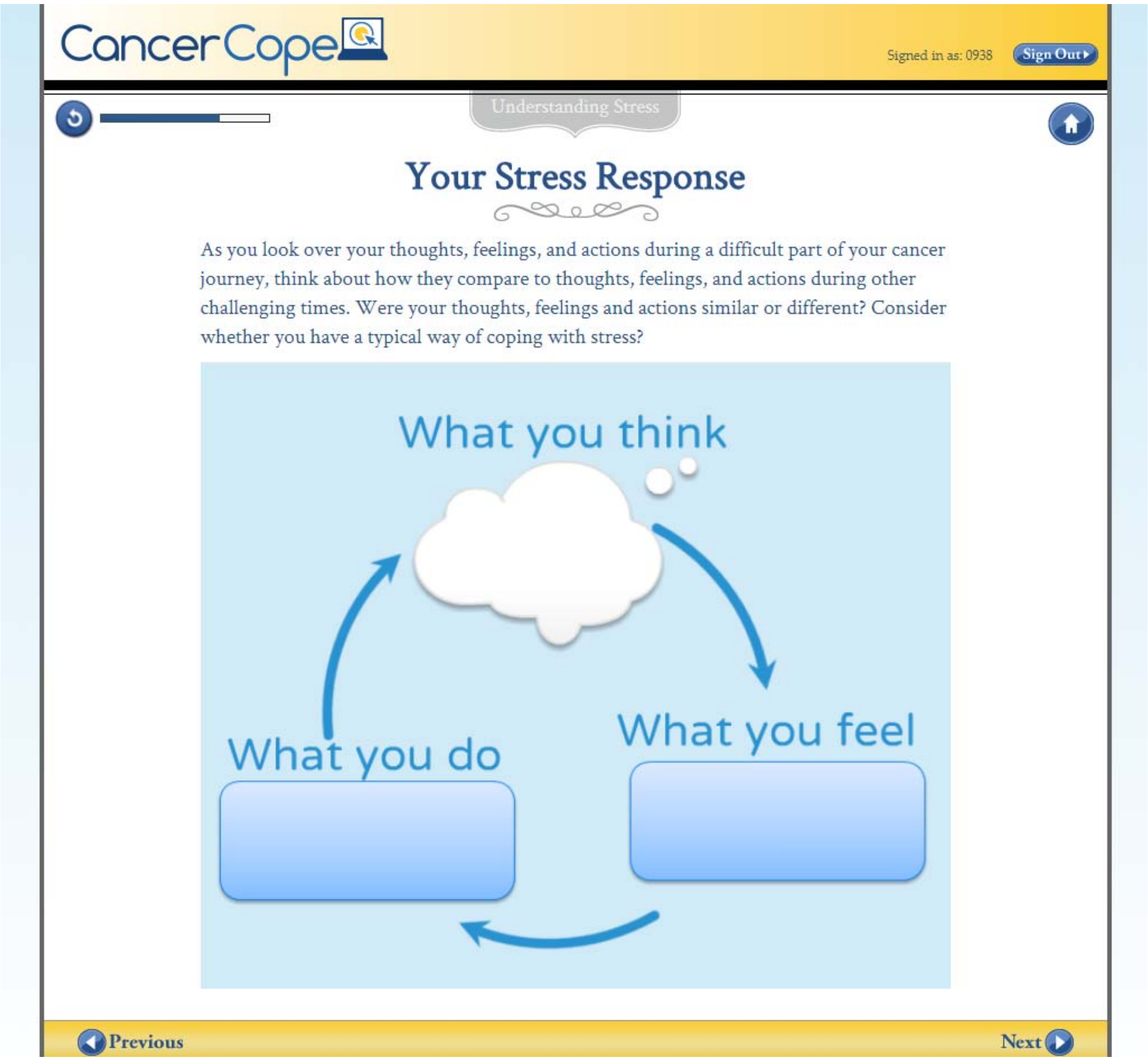

Your Values

As you enter your values below, click on the title of the circle if you want to see a reminder of helpful questions for that area. When you have completed each circle of values, click the SUBMIT button.

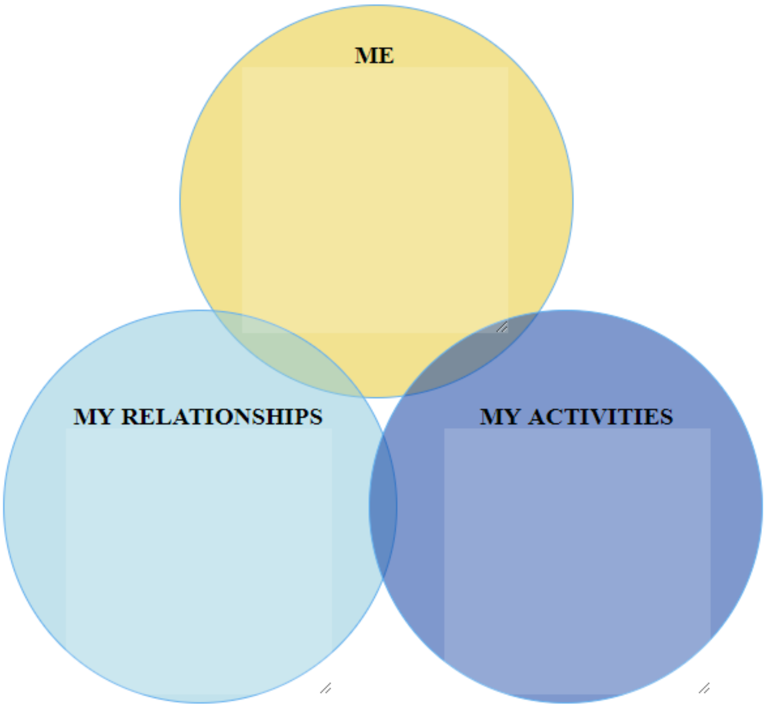

Submit

Steps for Successful Problem Solving

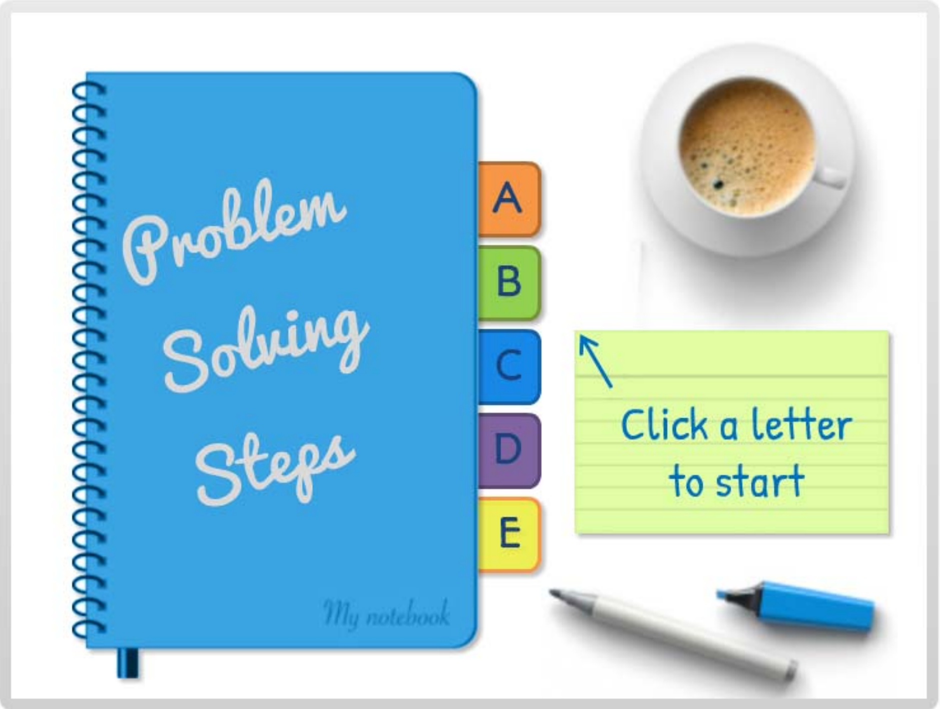

The cores contain stories of fictional characters to illustrate the different experiences of others on their cancer journey

CancerCope

Signed in as: 0938 Sign Out

Tackling Problems

Allan

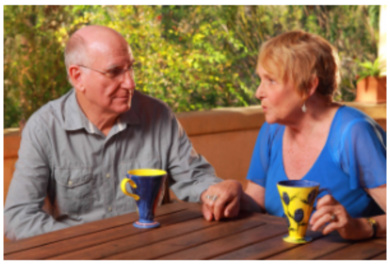

In the early days after his operation, sex was the last thing on Allan's mind. Initially he was focused on recovering from his surgery - getting his continence back and regaining his fitness.

A few months later his Urologist assured Allan it was time to try things out in the bedroom again, and sent Allan home with some samples of Viagra to try. Allan didn't have much success with the Viagra that night. After a couple more failed attempts, Allan just stopped trying.

He and Jude continue to be intimate in other ways but it's not the same. Allan has never felt any pressure from Jude but lately he feels as though their sex life (or lack of it) is the elephant in the room and it's time to seek help.

Click here to view Allan's attempt at working through the problem solving steps.

Back Next

© Copyright 2007-2015. BeHealth Solutions, LLC and the University of Virginia, Behavioral Health & Technology and Cancer Council Queensland. All rights reserved.
BeStudy Manager is a trademark of BeHealth Solutions, LLC. Copies, and/or reproduction of any kind, of the materials and/or information in this Website may not be made without the express permission of Cancer Council Queensland and BeHealth Solutions.

Moving Forward After Treatment

Click the photos of James and Margaret below to read about their experiences moving forward after treatment.

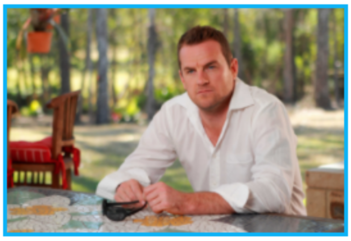
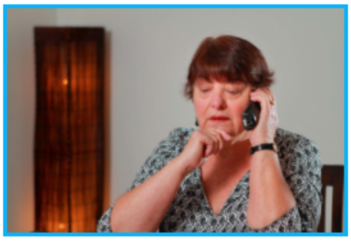

© Copyright 2007-2015. BeHealth Solutions, LLC and the University of Virginia, Behavioral Health & Technology and Cancer Council Queensland. All rights reserved.
BeStudy Manager is a trademark of BeHealth Solutions, LLC. Copies, and/or reproduction of any kind, of the materials and/or information in this Website may not be made without the express permission of Cancer Council Queensland and BeHealth Solutions.

CancerCope users receive tailored feedback based on distress scores and concerns

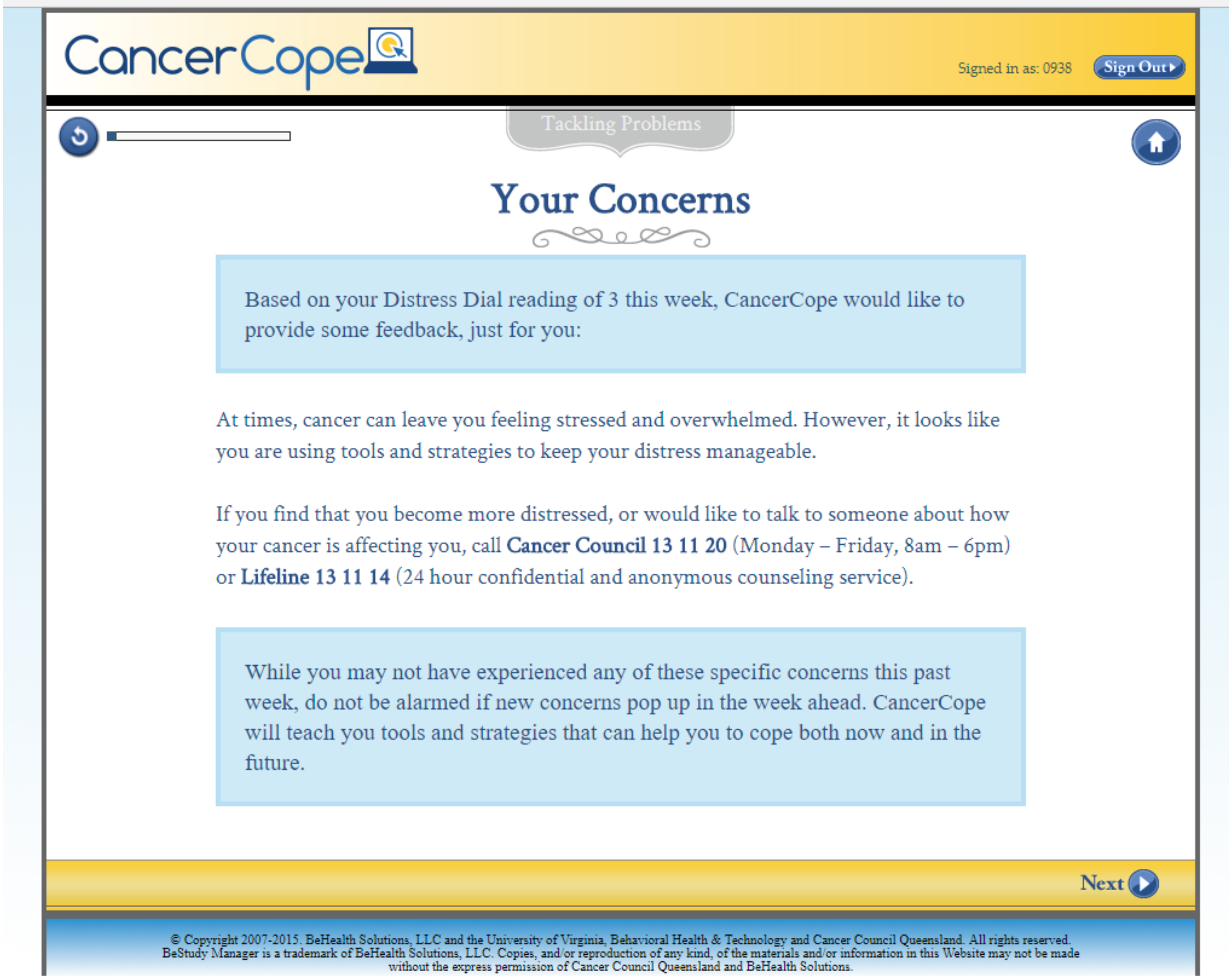

The CancerCope program included assigned behavioural homework supported by the interactive components of the website

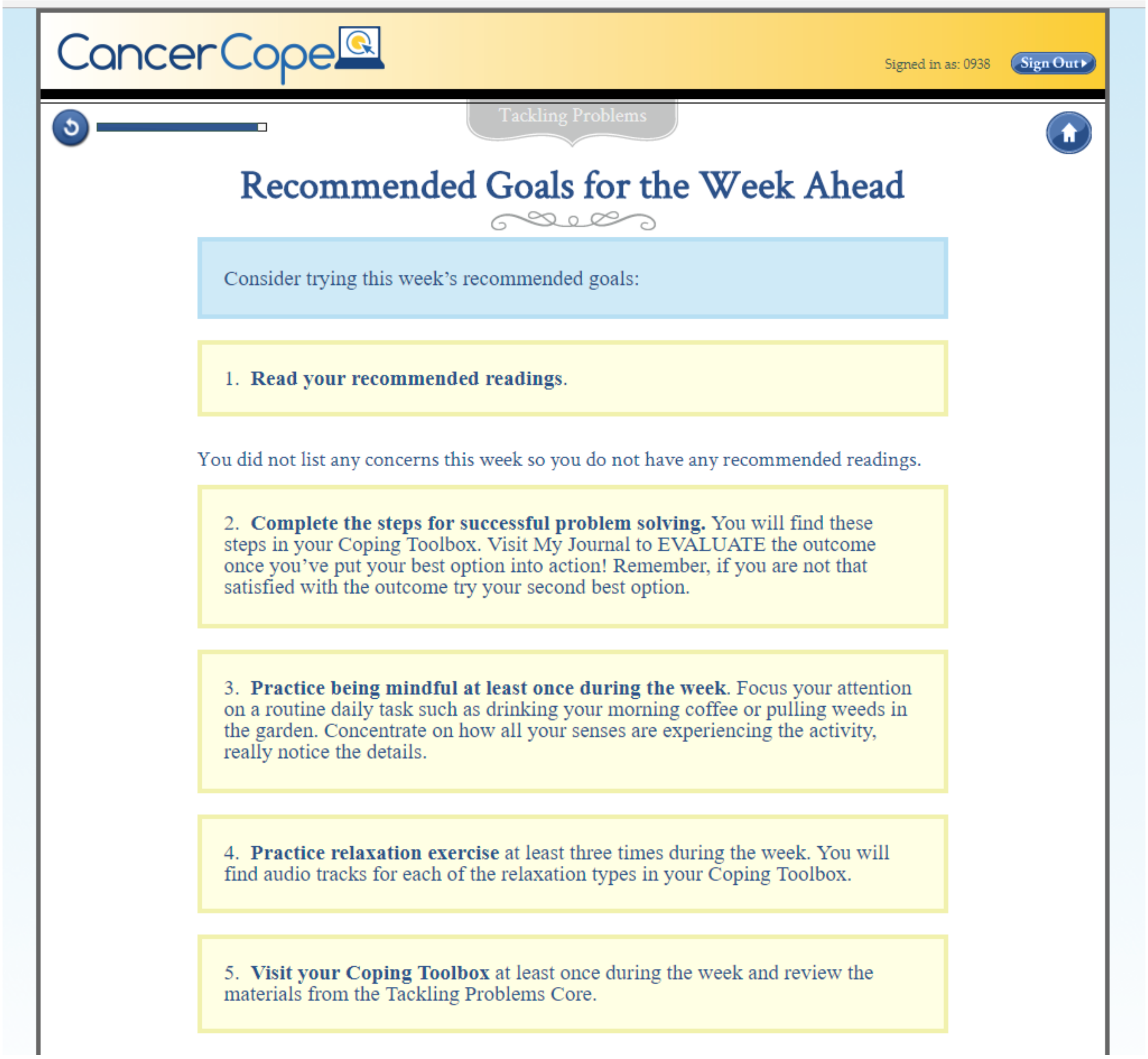

Users are encouraged to regularly review goals they have set previously to monitor their progress

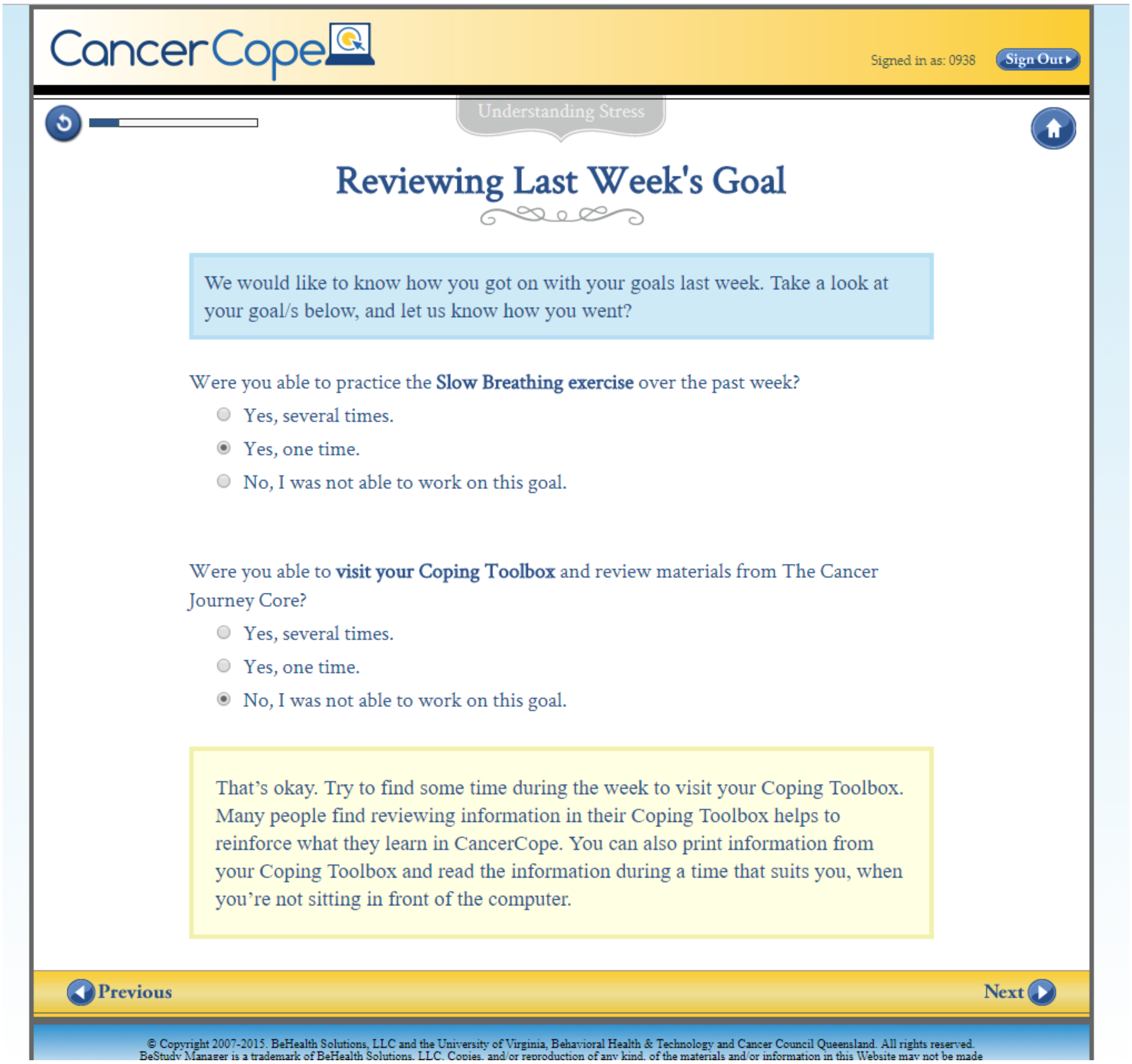

The program includes an electronic journal where participants can record their thoughts and feelings and reflect on their progress towards weekly goals.

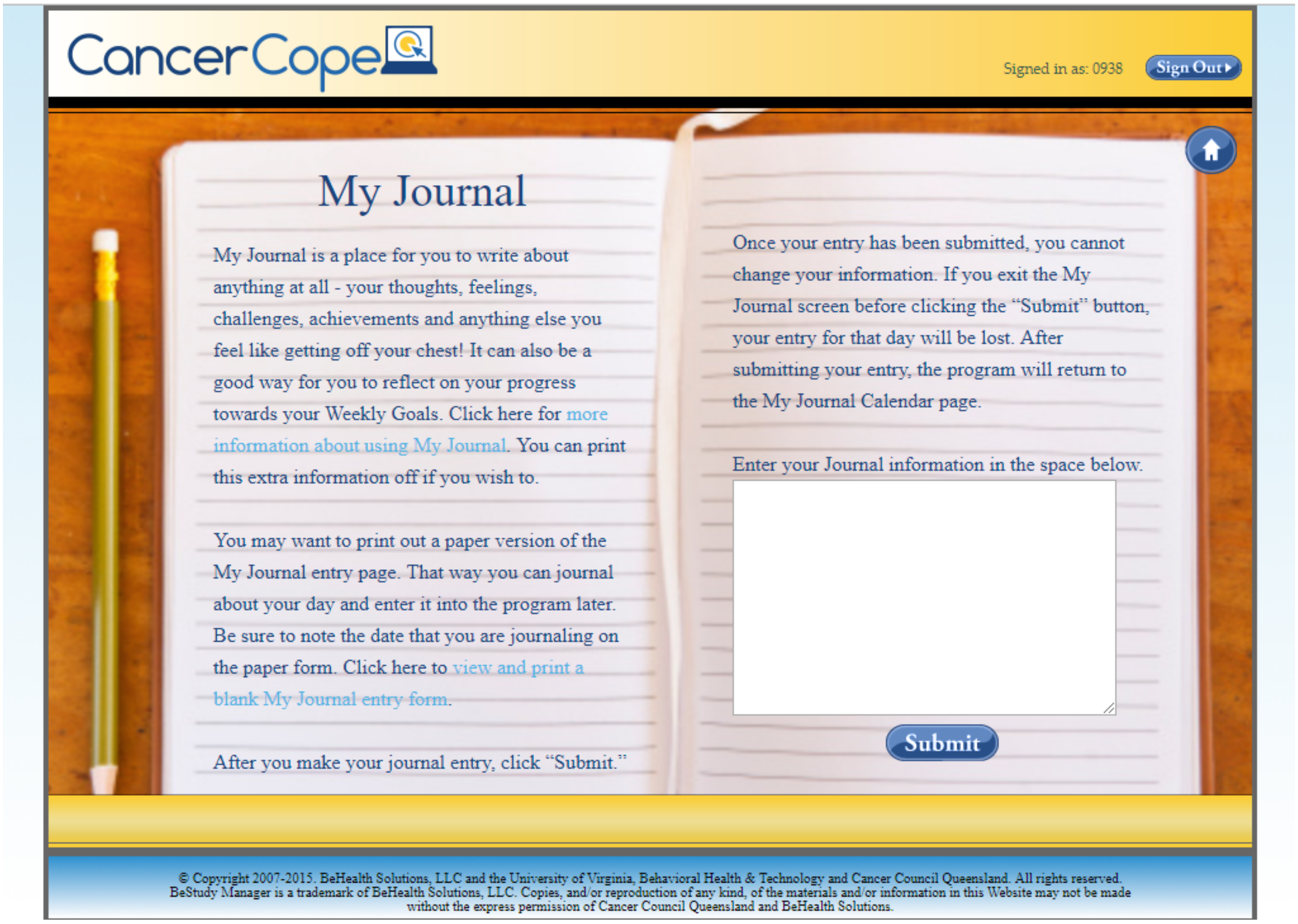

Participants are able to access their coping toolbox which contains information and resources from completed cores in one area

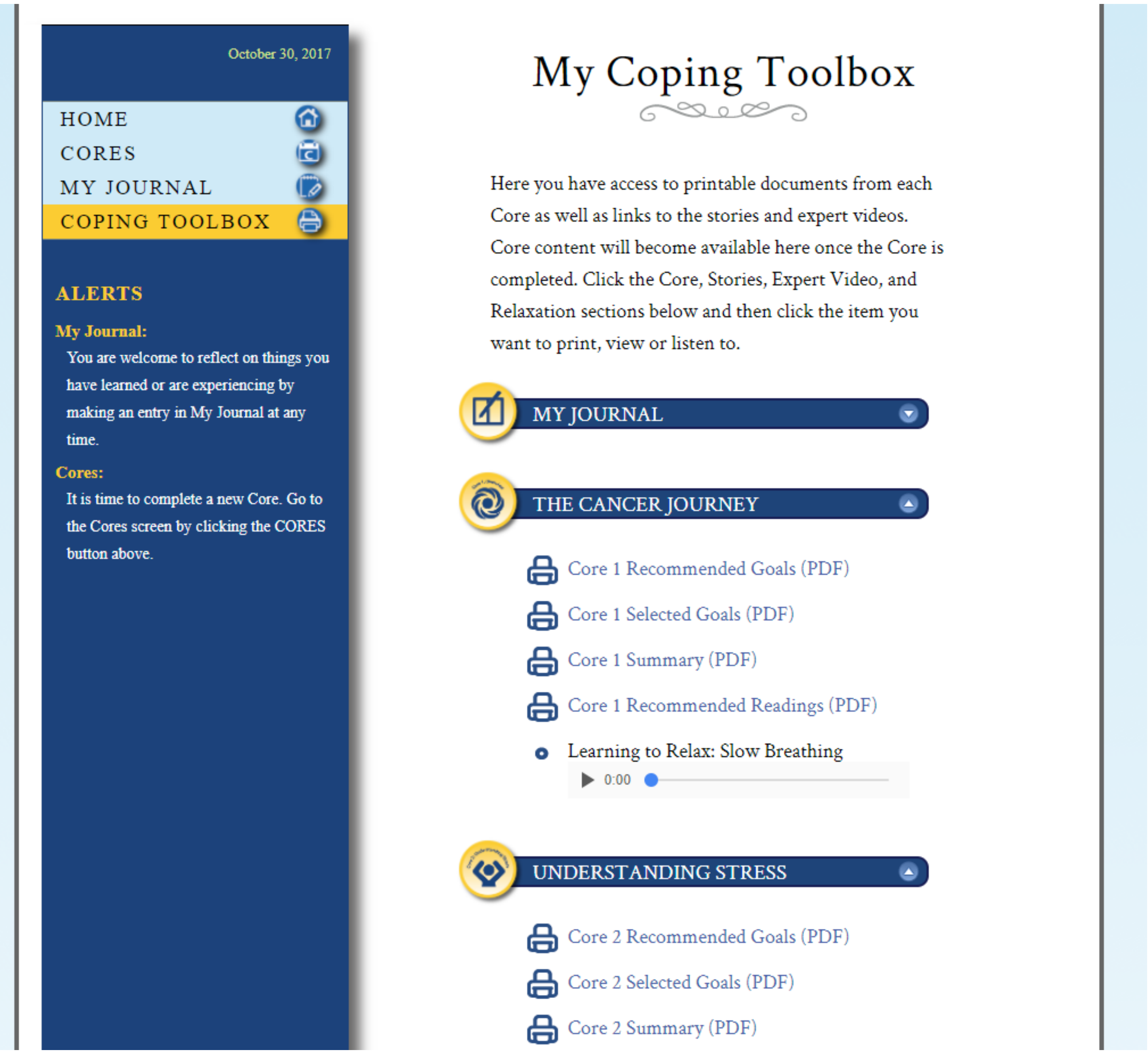

Supplement: Multimedia Appendix 1 [file jmir_v20i1e42_app1.pdf]
