# Supplementary material for: Web-Delivered Cognitive Behavioral Therapy for Distressed Cancer Patients: Randomized Controlled Trial
Source: J Med Internet Res. 2018 Jan 31;20(1):e42. doi: 10.2196/jmir.8850 (PMC5812983; doi:10.2196/jmir.8850)
Supplement: Multimedia Appendix 2 [file jmir_v20i1e42_app2.pdf]

Multimedia Appendix 2. Intention-to-treat analysis (baseline vs 2-month) for primary and secondary outcome scores using multiple imputation analysis (50 imputations).

	CancerCope				Patient Education				Test for Interaction[b]
	Baseline (n=79)		2 months (imputed) (n=79)		Baseline (n=84)		2 months (n=84)		
	$\bar{x}$	SD	$\bar{x}$	SD	$\bar{x}$	SD	$\bar{x}$	SD	*P*
**Variable**									
Psychological distress [a]	13.76	10.08	11.39	9.51	12.48	11.42	12.32	11.83	0.22
Cancer-specific distress [a]	30.25	17.97	26.08	18.09	29.74	17.13	**27.69**	18.64	0.43
Unmet needs									
Physical	37.72	26.27	35.91	26.53	43.04	27.78	39.08	28.05	0.68
Psychological	46.01	25.13	38.31	25.12	44.20	25.71	39.31	28.40	0.52
Health System and Information	25.66	19.81	24.16	22.11	25.54	20.33	27.97	26.70	0.45
Patient Care and Support	21.90	18.47	19.60	18.81	21.85	18.35	19.78	19.87	0.95
Sexuality	21.84	26.13	23.96	29.16	18.75	25.01	23.51	28.19	0.62
Health-related quality of life [a]	0.61	0.19	0.67	0.18	0.62	0.19	0.65	0.21	0.32
Posttraumatic growth [a]	47.63	23.00	51.63	21.60	42.52	24.14	49.32	22.57	0.54

[a] Psychological distress=BSI-18 Global Severity Index score; cancer-specific distress=IES total score; health-related quality of life=AQoL-8d utility score; posttraumatic growth=PTGI total score

[b] Interaction effects determined by hierarchical linear models for each outcome score between study groups (CancerCope and Patient Education) and time periods (Baseline, 2 months) Imputations derived using each of the outcome measures as Baseline, in addition to Age group and Sex.
