# Supplementary material for: Web-Delivered Cognitive Behavioral Therapy for Distressed Cancer Patients: Randomized Controlled Trial
Source: J Med Internet Res. 2018 Jan 31;20(1):e42. doi: 10.2196/jmir.8850 (PMC5812983; doi:10.2196/jmir.8850)
Supplement: Multimedia Appendix 3 [file jmir_v20i1e42_app3.pdf]

Multimedia Appendix 3. Sensitivity analysis (25% reduction in imputed values): intention-to-treat analysis (baseline vs 2-month) for primary and secondary outcome scores using multiple imputation analysis (50 imputations).

	CancerCope				Patient Education				Test for Interaction[b]
	Baseline (n=79)		2 months (imputed) (n=79)		Baseline (n=84)		2 months (n=84)		
	$\bar{x}$	SD	$\bar{x}$	SD	$\bar{x}$	SD	$\bar{x}$	SD	*P*
**Variable**									
Psychological distress [a]	12.19	9.21	10.07	8.48	11.16	10.56	11.09	11.29	0.18
Cancer-specific distress [a]	27.01	16.51	23.20	16.47	26.38	16.17	24.70	17.54	0.36
Unmet needs									
Physical	33.21	23.11	31.76	23.78	37.75	25.15	34.70	26.05	0.72
Psychological	40.84	23.43	33.83	22.48	39.14	24.33	34.97	26.68	0.45
Health System and Information	22.96	18.16	21.19	19.34	22.47	18.63	24.92	24.74	0.35
Patient Care and Support	19.43	16.20	17.35	16.89	18.97	16.35	17.50	18.13	0.85
Sexuality	20.12	24.72	21.29	26.35	16.87	22.96	20.68	25.18	0.55
Health-related quality of life [a]	0.54	0.19	0.59	0.19	0.55	0.19	0.56	0.19	0.25
Posttraumatic growth [a]	42.41	21.36	45.93	19.63	37.45	21.89	43.17	20.71	0.56

[a] Psychological distress=BSI-18 Global Severity Index score; cancer-specific distress=IES total score; health-related quality of life=AQoL-8d utility score; posttraumatic growth=PTGI total score

[b] Interaction effects determined by hierarchical linear models for each outcome score between study groups (CancerCope and Patient Education) and time periods (Baseline, 2 months) Imputations derived using each of the outcome measures as Baseline, in addition to Age group and Sex.
