# Supplementary material for: Web-Delivered Cognitive Behavioral Therapy for Distressed Cancer Patients: Randomized Controlled Trial
Source: J Med Internet Res. 2018 Jan 31;20(1):e42. doi: 10.2196/jmir.8850 (PMC5812983; doi:10.2196/jmir.8850)
Supplement: Multimedia Appendix 4 [file jmir_v20i1e42_app4.pdf]

Multimedia Appendix 4. Per-protocol analysis: intention-to-treat analysis (baseline vs 2-month) for primary and secondary outcome scores using multiple imputation analysis (50 imputations).

	>=3 cores accessed				Patient Education				Test for Interaction [b]	Effect size
	Baseline (n=22)		2 months (imputed) (n=22)		Baseline (n=84)		2 months (n=84)			
	$\bar{x}$	SD	$\bar{x}$	SD	$\bar{x}$	SD	$\bar{x}$	SD	*P*	d [CI]
**Variables**										
Psychological distress [a]	17.91	9.85	12.33	8.39	12.48	11.42	12.64	12.03	0.03	0.52 **[0.04, 0.99]**
Cancer-specific distress [a]	38.14	15.71	27.04	17.06	29.74	17.13	27.93	18.78	0.02	0.63 **[0.15, 1.10]**
Unmet needs										
Physical	37.50	23.49	41.25	27.09	43.04	27.78	41.19	28.41	0.47	**0.19 [0.00, 0.66]**
Psychological	54.89	22.87	36.32	24.56	44.20	25.71	39.59	29.08	**0.03**	0.56 **[0.08, 1.03]**
Health System and Information	37.40	26.93	24.96	23.08	25.54	20.33	28.63	27.12	0.05	0.47 **[0.01, 0.94]**
Patient Care and Support	30.91	20.27	22.93	21.72	21.85	18.35	19.88	20.27	0.28	0.27 **[0.00, 0.74]**
Sexuality	32.58	30.53	25.38	31.70	18.75	25.01	23.19	28.20	0.12	0.39 **[0.08, 0.85]**
Health-related quality of life [a]	0.55	0.18	0.65	0.16	0.62	0.19	0.64	0.21	0.10	0.40 [0.08, 0.87]
Posttraumatic growth [a]	48.95	22.11	59.62	17.54	42.52	24.14	48.99	23.28	0.52	0.15 [0.32, 0.62]

[a] Psychological distress=BSI-18 Global Severity Index score; cancer-specific distress=IES total score; health-related quality of life=AQOL-8d utility score; posttraumatic growth=PTGI total score.

[b] Interaction effects determined by hierarchical linear models for each outcome score between study groups (CancerCope >=3 cores accessed and Patient Education) and time periods (Baseline, 2 months) Imputations derived using each of the outcome measures as Baseline, in addition to Age group and Sex
