# Supplementary material for: Web-Delivered Cognitive Behavioral Therapy for Distressed Cancer Patients: Randomized Controlled Trial
Source: J Med Internet Res. 2018 Jan 31;20(1):e42. doi: 10.2196/jmir.8850 (PMC5812983; doi:10.2196/jmir.8850)
Supplement: Multimedia Appendix 5 [file jmir_v20i1e42_app5.pdf]

Multimedia Appendix 5. Sensitivity analysis (25% reduction in imputed values): per-protocol analysis (baseline vs 2-month) for primary and secondary outcome scores using multiple imputation analysis (50 imputations).

	>=3 cores accessed				Patient Education				Test for Interaction [b]	Effect size
	Baseline (n=22)		2 months (imputed) (n=22)		Baseline (n=84)		2 months (n=84)			
	$\bar{x}$	SD	$\bar{x}$	SD	$\bar{x}$	SD	$\bar{x}$	SD	*P*	d [CI]
**Variables**										
Psychological distress [a]	17.25	9.28	11.93	7.96	11.15	10.56	11.33	11.37	0.02	0.56 [0.08, 1.03]
Cancer-specific distress [a]	37.16	15.36	26.22	16.54	26.40	16.17	24.88	17.59	0.01	0.70 [0.22, 1.17]
Unmet needs										
Physical	36.82	23.73	40.31	26.70	37.75	25.15	36.28	25.94	0.47	0.19 [0.29, 0.66]
Psychological	53.38	22.18	35.36	23.91	39.13	24.33	35.18	27.05	0.01	0.63 [0.15, 1.11]
Health System and Information	35.95	24.75	24.35	22.41	22.47	18.63	25.42	24.92	0.05	0.49 [0.02, 0.97]
Patient Care and Support	29.49	18.38	22.20	21.00	18.97	16.34	17.57	18.36	0.24	0.30 [0.18, 0.77]
Sexuality	32.58	30.53	25.00	31.40	16.87	22.96	20.44	25.22	0.08	0.44 [0.04, 0.91]
Health-related quality of life [a]	0.55	0.19	0.64	0.17	0.55	0.19	0.56	0.20	0.06	0.47 [0.00, 0.95]
Posttraumatic growth [a]	48.07	21.81	58.34	17.72	37.45	21.89	42.92	21.22	0.38	0.22 [0.00, 0.69]

[a] Psychological distress=BSI-18 Global Severity Index score; cancer-specific distress=IES total score; health-related quality of life=AQOL-8d utility score; posttraumatic growth=PTGI total score.

[b] Interaction effects determined by hierarchical linear models for each outcome score between study groups (CancerCope >=3 cores accessed and Patient Education) and time periods (Baseline, 2 months) Imputations derived using each of the outcome measures as Baseline, in addition to Age group and Sex
